# Evaluation of the partners in research course: a patient and researcher co-created course to build capacity in patient-oriented research

**DOI:** 10.1186/s40900-021-00316-8

**Published:** 2021-10-30

**Authors:** Melissa Courvoisier, Richelle Baddeliyanage, Linda Wilhelm, Lorraine Bayliss, Sharon E. Straus, Christine Fahim

**Affiliations:** 1 myBlueprint, 310 Davenport Rd, Toronto, ON M5R 1K6 Canada; 2 Knowledge Translation Program, Li Ka Shing Knowledge Institute, St. Michael’s Hospital, 209 Victoria St., Toronto, ON M5B 1T8 Canada; 3 Dalla Lana School of Public Health, University of Toronto, 155 College St. Room 500, Toronto, ON M5T 3M7 Canada; 4 The Canadian Arthritis Patient Alliance, Midland, Canada; 5 Transdisciplinary Understanding and Training On Research (TUTOR), Centre for Studies in Family Medicine, Western University, 1151 Richmond St., London, ON N6A 3K7 Canada; 6 Department of Medicine, Faculty of Medicine, University of Toronto, 1 King’s College Circle, Medical Sciences Building, Toronto, ON M5S 1A8 Canada

**Keywords:** Patient and public involvement, Patient-oriented research, Patient engagement, Course evaluation, Co-creation, PPI training

## Abstract

**Background:**

In the past decade, patient-oriented research (POR) has been at the forefront of healthcare research in Canada because it has the potential to make research more meaningful and relevant to patient needs. Despite this growing emphasis on and expectation to conduct POR, there is limited guidance about how to apply POR in practice. To address this capacity building need, the Knowledge Translation (KT) Program and patient partners co-designed, delivered, and evaluated *Partners in Research* (PiR), a 2-month online course for patients and researchers to collectively learn how to conduct and engage in POR.

**Methods:**

PiR was delivered to 4 cohorts of patients and researchers between 2017 and 2018. For each cohort, we evaluated the impact of the course on participants’ knowledge, self-efficacy, intentions, and use of POR using surveys at 3 time points: baseline, post-course and 6-months post-course. We also monitored the process of course design and delivery by assessing implementation quality of the PiR course. Participants were asked to rate their satisfaction with course format, course materials, quality of delivery and their level of engagement via a 7-point Likert scale in the post-course survey.

**Results:**

A total of 151 participants enrolled in the PiR course throughout the 4 cohorts. Of these, 49 patients and 33 researchers (n = 82 participants) consented to participate in the course evaluation. Process and outcome evaluations collected over a 9-month period indicated that participation in the PiR course increased knowledge of POR concepts for patients (*p* < .001) and for researchers (*p* < .001) from pre-course to post-course timepoints. Likewise, self-efficacy to engage in POR increased from baseline to post-course for both patients (*p* < .001) and researchers (*p* < .001). Moreover, participants reported high levels of satisfaction with content, delivery and interactive components of the course.

**Conclusions:**

The PiR course increased capacity in POR for both researchers and patients. This work enhances our understanding of how to design useful and engaging education opportunities to increase patient and researcher capacity in POR.

**Supplementary Information:**

The online version contains supplementary material available at 10.1186/s40900-021-00316-8.

## Plain English Summary

Patient-oriented research (POR) is the meaningful inclusion of patients as *active contributing partners* in research. In meaningful POR, patients are engaged and valued as contributory members of the research team throughout the research process, from developing research questions, participating in research activities, to sharing new research findings. In Canada, POR is recognized as an important component of research and many healthcare institutions aim to engage patients in their work. However, there is little guidance on how patients and researchers can effectively participate as equal partners in research activities. To address the need for POR education opportunities, we launched *Partners in Research* (PiR), a 2–month online course for patients and researchers to learn how to conduct and engage in POR. This paper describes the design, delivery, and evaluation of the PiR course, with a focus on the following objectives:

1. To assess the impact of the PiR course on participant knowledge, confidence, intentions and use of POR
2. To determine the quality of course design and delivery and its ability to achieve learning objectives
3. To identify factors that limit and facilitate participation in POR

The findings from the evaluation show that the PiR course was effective in increasing knowledge of, and confidence in POR concepts. In addition, the course was well-received, with participants reporting a high level of engagement in course content and activities. Overall, this work supports our understanding of how to design useful and engaging opportunities to increase patient and researcher capacity in POR.

**Supplementary Information:**

The online version contains supplementary material available at 10.1186/s40900-021-00316-8.

## Background

In the past decade, patient-oriented research (POR) has been at the forefront of healthcare research in Canada [1]. POR aims to meaningfully include patient perspectives and priorities in research design, execution, analysis, dissemination, implementation and evaluation to ensure research is aligned with patient needs [1]. It is important to emphasize that POR aims to engage patients *meaningfully* in the research process as opposed to tokenistic engagement in research. Tokenistic engagement can be characterized as involving patients as mere signatories on research grants or publication submissions, while not actively capturing and including patient ideas, needs and experiential insights in the research process or outcomes. Conversely, in meaningful POR, patients are engaged as active, contributory members of the research team and their insights are used to shape the direction, methods, analysis, and/or outcomes of the research. POR provides an opportunity for patient collaborators to occupy an important and autonomous role within the evidence to practice continuum. Such involvement can ensure that public research dollars are meaningfully spent on patient-identified priorities, which may result in improved uptake of evidence-based recommendations, thereby reducing research waste [2, 3].

Without patient perspectives, there is increased potential of misalignment of research goals and outcomes between patients and researchers [3, 4]. The potential of POR to improve health systems and practices is now recognized by research funding organizations globally, including federal funding bodies such as the Canadian Institutes of Health Research, who commissioned the Strategy for Patient-Oriented Research (SPOR) [5] in a pan-Canadian effort to increase POR.

Despite this growing emphasis on and expectation to conduct POR, there is limited guidance about how to apply POR in practice [6–9]. Furthermore, opportunities for patients and researchers to build capacity in POR in order to support research collaborations are limited [10]. To address this capacity building need, the Knowledge Translation (KT) Program and patient partners co-designed, administered, and evaluated *Partners in Research* (PiR), a 2-month online course for patients and researchers to collectively learn how to conduct and engage in POR.

In this paper, we describe the design, delivery and evaluation of the PiR course. The primary objective of the evaluation was to describe the impact of the PiR course on participant (patient and researcher) knowledge about POR, self-efficacy to engage in POR (i.e., confidence in one’s ability to effectively practice POR), intentions to use, and use of POR. The secondary objectives were to determine the implementation quality of the PiR course (i.e., how well the course was delivered) and identify participant-reported barriers and facilitators to engaging in POR.

## Methods

A steering committee that included a patient partner, an education specialist, an evaluation expert, and 2 scientists met quarterly to oversee the direction and operationalization of this study.

### Designing the PiR course

The course design was informed by various learning theories and best practices in education including transformative and social learning theories [11–13], critical pedagogy and adult learning theory [14, 15], as well as online education models such as the Community of Inquiry Model and the Teaching Excellence Competencies Model [16–20]. The Canadian Institutes of Health Research SPOR Patient Engagement Framework was also used to guide the course design [5]. This framework outlines core principles of POR such as inclusiveness, support, mutual respect, and co-building. These guiding principles were used to meaningfully engage patient and research partners in the co-design, delivery and evaluation of the PiR course.

We created a working group composed of two patient partners, two research partners, an adult education specialist and a research assistant. Patient partners who sat on the steering committee or the working group were recruited from within our existing network of patient partners, established via previous research activities led by our research team.

Over a four-month period, the working group convened to operationalize the course design, delivery, and evaluation. The working group met monthly using an online meeting platform (Cisco Webex) and communicated routinely via email and telephone. Patient and researcher partners provided direction on the optimal course content, structure and needed learning supports. The course content focused on defining meaningful POR and distinguishing it from tokenistic engagement; common barriers and facilitators to POR, and the skills and attitudes that support POR in practice. The group iteratively revised each course offering based on course participant feedback. An additional file of the course syllabus outlines a complete overview of the PiR course components (see Additional file 1).

### Delivering the PiR course

PiR was a 2-month, online course that included 4 bi-weekly real-time webinars, self-assessment quizzes, problem-based learning scenario (PBLS) assignments, and online group coaching. Each PiR module topic was co-presented in real-time by a patient and a researcher both of whom had expertise in POR. This effectively provided participants with the opportunity to engage in dialogue with POR experts and fellow learners. Each module also included discussion boards housed on the online learning platform, Canvas, that were co-facilitated by multiple researcher and patient coaches with POR experience. Course presenters (patients and researchers with expertise in POR who delivered real-time online presentations) and coaches (patients and researchers with expertise in POR who facilitated asynchronous online discussions) were recruited through various channels including the KT Program network, patient collaborator networks, and the PiR course participants who had successfully completed the course. Additionally, presenters and coaches of each course iteration tailored the pre-designed webinar slides and group discussion forums to include their own POR lived experiences and insights.

Coaches were provided with a comprehensive handout on coaching objectives and best practices and were connected to a course facilitator who was available to support the development of discussion posts and responses. Presenters were also supported by a course facilitator to prepare and deliver their presentations. A facilitator met with each presenter to discuss the overall topic to be presented, to share pre-developed slides and key talking points, and to encourage presenters to tailor the presentation to highlight their own experiences. Additionally, the course facilitator scheduled and facilitated meetings to connect the researcher and patient co-presenters to foster social connection and meaningful collaboration.

In the 2-week intervals between each webinar, participants were expected to complete small group PBLS assignments and participate in online group discussion boards. The PBLS assignments provided opportunities for small groups of patients and researchers to collectively reflect on common POR challenges and identify potential solutions. Similarly, the online discussion boards provided opportunities for participants to engage in discussions about barriers and facilitators to engaging in POR.

### Participant recruitment

PiR was offered to four cohorts of patients, family members, and caregivers (hereafter referred to as patients for brevity) and healthcare researchers between May 2017 and December 2018. We invited a maximum of 40 participants (20 patients, 20 researchers) to participate in each cohort [21]. This number of participants was large enough to allow for a multiplicity of ideas and experiences to contribute to the co-production of knowledge about POR while small enough to facilitate meaningful participant engagement.

Course recruitment materials were disseminated via the KT Program’s, research organizations’ and patient partners’ networks (e.g., the Patient Advisors Network [22]). Participants were eligible to participate if they were either a healthcare researcher or a patient or a patient’s family member/caregiver. Both researcher and patient participants were not required to have POR experience, but were required to submit an application to attend the course that outlined their learning goals and interests in POR. The course was open to participants from across Canada, but recruitment was prioritized in Ontario. PiR course participants were invited to participate in the evaluation study via email invitation during the PiR course onboarding process. Those who agreed to participate in the course evaluation were emailed an online study information sheet and consent to participate.

### Study design

We used a longitudinal, uncontrolled before and after study design to conduct a process and outcome evaluation of the PiR course. Patient partners contributed to the evaluation design and were consulted in the preparation of the study protocol. The course evaluation was informed by Kirkpatrick’s four-level training evaluation model [23].

### Study outcomes

The primary outcomes of this study were to determine the perceived impact of the PiR course on participants’ knowledge, self-efficacy, intentions to use and use of POR. These outcomes were evaluated using online surveys, which were administered at three time points: baseline (i.e., before the start of the course), immediately post-course, and six months after course completion. Surveys were designed to measure participants’ self-identified knowledge of POR concepts (17 questions), self-efficacy to engage in POR (17 questions), intentions to engage in POR (8 questions) and use of POR (3 questions). Participants rated their level of agreement with each statement on a 7-point likert scale. Open-ended questions were included to gather additional information on participant-level outcomes. The COM-B Behaviour Change Theory [24] and the Theoretical Domains Framework (TDF) [25] were used to inform survey questions related to knowledge and self-efficacy of POR engagement. COM-B is a widely used model of behavior change identifying factors that need to be present for behavior change to occur [24]. The TDF is a comprehensive framework used to identify determinants of behavior [25]. Additionally, Légaré’s 12 item intention assessment scale was used to design survey questions about participants’ intentions to engage in POR [25]. Légaré’s scale is a theory-based instrument that shows adequate validity and reliability [26].

The secondary outcome of implementation quality assessed reach of the PiR course, dose of the PiR course components, and participant responsiveness. These indicators were informed by the Durlak and DuPre framework, which indicates that the quality of implementation affects the intended outcome and therefore relevant indicators should be used to assess intervention implementation quality [27]. Course reach was measured by the number of patients and researchers who participated in each cohort. Participant responsiveness (or level of engagement with the course) was reported descriptively as number of responses to each discussion board post, and participant satisfaction scores. Participants were asked to rate their satisfaction with course format (6 questions), course materials (7 questions), quality of delivery (9 questions), and their level of engagement (i.e., responsiveness, 3 questions) via a 7-point Likert scale in a post-course survey. Open-ended questions were also included to identify opportunities to improve the course and determine how the course compared to other POR training. Finally, via survey, participants were asked to describe barriers and facilitators to engaging in POR. These secondary outcomes align with Kirkpatrick’s training evaluation model [23]. An additional file outlines the survey items in further detail (see Additional file 2).

### Data collection

Outcome data were collected via surveys as well as a review of course administrative documents and discussion boards. All surveys were delivered online using Qualtrics software [28]. To optimize survey response rate, the Total Design Method (TDM) was used, whereby reminders were delivered to non-responders at 1-week, 3-weeks and 7-week intervals [29]. Accordingly, participants had a total of 7-weeks to respond to the survey at each timepoint. An additional file further outlines the data sources (see Additional file 3).

### Data analysis

Survey data from all 4 cohorts were aggregated and analysis was stratified by participant type (patients vs. researchers). Analysis was conducted using Stata 16 [30]. Descriptive statistics were used to summarize survey scores for participant groups across all 3 survey time points. Given the data were not normally distributed, a non-parametric two-sample Wilcoxon rank-sum test was used to determine whether there was a statistically significant difference in primary outcomes between time points. In addition, a two-sample Wilcoxon rank-sum test was completed to determine if patient and researcher groups significantly differed in primary outcomes scores across time points.

Open-ended survey responses were exported to Microsoft Excel software for qualitative analysis. Survey responses were aggregated across cohorts and stratified by participant type. Each timepoint was analyzed separately and thematic differences between timepoints were recorded. Data were double coded independently by two researchers; coding discrepancies were resolved through consensus meetings. The complete coding framework is presented in an additional file, but in brief, participant responses were coded into the primary outcome constructs of knowledge, self-efficacy, intentions and use of POR and secondary outcome constructs of implementation quality and participant responsiveness (see Additional file 4). Participant-reported barriers and facilitators to POR were analyzed to identify emergent, common themes. Illustrative quotes were abstracted to provide rich descriptions of participant feedback.

## Results

### Participants

A total of 151 participants, including 74 patients and 77 health researchers participated in the 4 PiR course cohorts (Table 1). Of the 151 enrolled participants, 49 patients and 33 researchers (n = 82 participants) consented to participate in the course evaluation. The survey response rate at baseline for patients was 85.7% (n = 42) and for researchers was 81.8% (n = 27); at post course was 71.4% (n = 35) for patients and 78.8% (n = 26) for researchers; and at 6-months following the course was 63.2% (n = 31) for patients and 72.7% (n = 24) for researchers. Although survey response rates decreased over the three time points for both participant groups, researchers had noticeably less non-responders than patients.

**Table 1:** Reach of the PiR course and evaluation by participant group

Cohort	Course			Cohort	Evaluation			
	Patients	Healthcare researchers	Total		Timepoint	Patients	Healthcare researchers	Total
1 (Spring 2017)	18	18	36	1 (Spring 2017)	BL	8	7	15
					PC	8	6	14
					6M	8	7	15
2 (Fall 2017)	17	23	40	2 (Fall 2017)	BL	12	8	20
					PC	11	7	18
					6M	9	6	15
3 (Spring 2018)	19	19	38	3 (Spring 2018)	BL	10	4	14
					PC	10	5	15
					6M	6	3	9
4 (Fall 2018)	20	17	37	4 (Fall 2018)	BL	12	8	20
					PC	6	8	14
					6M	8	8	16
Total	74	77	151	Total	BL	42	27	69
					PC	35	26	61
					6M	31	24	55

*BL* Baseline timepoint, *PC* post-course timepoint, *6M* 6-month timepoint

### Primary outcomes

#### Change in knowledge and self-efficacy over time

Participants’ knowledge and self-efficacy for POR was reported as moderate at baseline (Table 2). There was a significant increase in knowledge of POR concepts for patients (Z = 5.41, *p* < 0.001) and for researchers (Z = 4.76, *p* < 0.001) from baseline to post-course (Table 2, Additional file 5). Similarly, the patient group had a significant increase in knowledge between post-course to 6-months post-course (Z = 2.27, *p* = 0.02). However, there was no significant difference in knowledge between post-course to 6-months post-course for researchers (Z = 0.68, *p* = 0.50). Likewise, self-efficacy to engage in POR increased from baseline to post-course for both patients (Z = 5.61, *p* < 0.001) and researchers (Z = 4.87, *p* < 0.001, yet no significant differences were observed in either group from post-course to 6-months post-course. Results comparing patient and researcher scores indicate there was no significant differences in knowledge and self-efficacy between the two participant groups (Table 3). The open-ended survey data reflected these trends; participants stated that the PiR course contributed to increased knowledge in POR concepts and increased self-efficacy in practicing POR. Following the course, participants felt they were more aware of the spectrum of POR activities and were better equipped to understand how to avoid tokenistic engagement.

**Table 2:** Differences in knowledge, self-efficacy, intentions and behaviour between study timepoints

	Patients				Researchers			
	Mean (SD)^a^	Median [95% CI]^a^	Test of difference between BL and PC^b^	Test of difference between PC and 6 Mb	Mean (SD)^a^	Median [95% CI]^a^	Test of difference between BL and PC^b^	Test of difference between PC and 6 Mb
Knowledge								
Baseline	4.48 (1.05)	4.53 (4.21, 5.07)	z score = 5.41	z score = 2.27	4.67 (1.14)	4.61 (4.07, 5.01)	z score = 4.76	z score = 0.68
Post course	5.74 (0.63)	5.72 (5.62, 5.99)	*p* < 0.001*	*p* = 0.02*	6.16 (0.44)	6.00 (5.89, 6.42)	*p* < 0.001*	*p* = 0.50
6 M	6.08 (0.51)	6.15 (5.78, 6.29)			6.23 (0.51)	6.27 (5.99, 6.48)		
Self-efficacy								
Baseline	4.21 (1.04)	4.25 (3.74, 4.71)	z score = 5.61	z score = 1.75	4.29 (1.25)	4.16 (3.65, 4.99)	z score = 4.87	z score = 0.29
Post course	5.58 (0.58)	5.55 (5.37, 5.88)	*p* < 0.001*	*p* = 0.08	4.97 (0.52)	5.96 (5.81, 6.15)	*p* < 0.001*	*p* = 0.77
6 M	5.85 (0.54)	5.85 (5.60, 6.18)			6.01 (0.54)	5.94 (5.72, 6.22)		
Intentions								
Baseline	6.01 (6.01)	6.06 (5.75, 6.38)	z score = 0.68	z score = 2.35	6.01 (0.74)	6.13 (5.63, 6.57)	z score = 0.15	z score = 0.20
Post course	5.95 (0.62)	6.00 (5.64, 6.25)	*p* = 0.50	*p* = 0.02*	6.07 (0.61)	6.14 (5.92, 6.45)	*p* = 0.88	*p* = 0.85
6 M	6.17 (0.87)	6.38 (6.19, 6.47)			6.12 (0.59)	6.20 (5.87, 6.50)		
Behaviour								
Baseline	4.83 (1.83)	5.00 (4.33, 6.00)	z score = 0.66	z score = 0.78	4.19 (1.80)	4.13 (3.00, 5.45)	z score = 2.12	z score = 1.36
Post course	5.10 (1.73)	5.67 (5.00, 6.00)	*p* = 0.51	*p* = 0.43	5.21 (1.64)	5.83 (4.67, 6.33)	*p* = 0.03*	*p* = 0.17
6 M	5.26 (1.90)	6.00 (5.16, 6.50)			5.73 (1.47)	6.17 (5.44, 6.78)		

*BL* Baseline timepoint, *PC* post-course timepoint, *6 M* 6-month timepoint, *SD* standard deviation

^a^Descriptive statistics: mean score and standard deviation, median score and 95% confidence intervals

^b^Two-sample Wilcoxon rank-sum test (Z-score and *p*-value)

^*^Denotes a significant result

**Table 3:** Differences in knowledge, self-efficacy, intentions and behaviour between patients and researchers across study timepoints

	Mean Δ BL to PC (PC – BL)* (SD)^a^		Test of difference between patient and researcher^b^	Mean Δ PC to 6 M (6 M – PC)** (SD)^a^		Test of difference between patient and researcher^b^
	Patients	Researchers		Patients	Researchers	
Knowledge	1.50 (1.31)	1.58 (0.83)	Z score = *p* = 0.56	0.20 (0.50)	0.08 (0.41)	*p* = 0.68
Self-efficacy	1.60 (0.96)	1.72 (0.89)	*p* = 0.69	0.15 (0.43)	0.04 (0.39)	*p* = 0.49
Intentions	− 0.04 (0.81)	0.21 (0.57)	*p* = 0.12	0.21 (0.65)	0.03 (0.46)	*p* = 0.26
Behaviour	0.50 (1.93)	0.87 (1.61)	*p* = 0.16	0.04 (1.42)	0.48 (1.04)	*p* = 0.58

*BL* Baseline timepoint, *PC* post-course timepoint, *6M* 6-month timepoint, *SD* standard deviation

^a^Descriptive statistics: mean score and standard deviation

^b^Two-sample Wilcoxon rank-sum test (Z-score and *p*-value)

^*^Includes only participants who completed BL and PC

^**^Includes only participants who completed PC and 6M

#### Intentions to engage in POR

At all three time-points, participants had high intentions to engage in POR with mean scores greater than 5.95. There was no significant change in intentions to engage in POR among participants between the baseline and post-course time points (Table 2, Additional file 5). Patients’ intentions to engage in POR was observed to have a statistical increase in mean score between post-course to 6-months post-course (Z = 2.35, *p* = 0.02). However, researchers’ intentions remained unchanged across time points (Z = 0.15, *p* = 0.88). There was no significant difference in intention to use POR across participant groups (Table 3). Open-ended survey responses reinforced that participants’ intentions to expand capacity in POR were high at baseline and remained high after the course.

#### Use of POR

At baseline, patients and researchers reported having moderate experience engaging in POR with reported mean scores of 4.8 (*SD* = 1.8) and 4.2 (*SD* = 1.8) respectively (Table 2). While patient participants did not demonstrate a significant improvement in engaging in POR following the course (Z = 0.66, *p* = 0.51), researchers’ reported use of POR continued to improve significantly from baseline to the post-course time point (Z = 2.12, *p* = 0.03) (Table 2, Additional file 5). There was no observed difference in use of POR scores from post-course to 6-months post course for both patients (Z = 0.78, *p* = 0.43) and researchers (Z = 1.36, *p* = 0.17). Moreover, use of POR scores did not significantly differ between patient and researcher groups from baseline to post-course (*p* = 0.16) and post-course to 6-months post-course (*p* = 0.58) (Table 3). Qualitative findings indicated that patients were actively seeking participation in POR but were finding it difficult to identify meaningful engagement opportunities, and recommended patient-oriented researcher groups foster additional opportunities for patient involvement in research. Conversely, researchers reported high levels of engagement in POR projects, most notably during the post-course period.

### Secondary outcomes

#### Course format

Patients and researchers were satisfied with the course format (*M* = 5.90, *SD* = 0.96, and *M* = 6.05, = 0.59, respectively). Participants expressed their preference for the interactive components of the course and the ability to connect and learn from patient and researcher experts in POR. The course format was perceived by participants to be effective in establishing a collaborative and stimulating learning environment.

#### Quality of delivery

Participants perceived the quality of course delivery to be high (*M* = 6.02, *SD* = 0.85 for patients and *M* = 6.26, *SD* = 0.58 for researchers). Participants reported that the course components were well organized and structured to stimulate co-learning. Additionally, course facilitators were perceived to encourage engagement and provide a supportive and accessible platform to gain knowledge and self-efficacy in POR.

#### Participant responsiveness

Participant satisfaction was high across all cohorts (*M* = 6.13, *SD* = 0.98 for patients and *M* = 6.30, *SD* = 0.52 for researchers). Participants enjoyed the course structure, opportunities for discussion, and inclusion of diverse perspectives through patient and researcher presenters. For example, one patient participant noted that.

it was nice to hear how the level of acceptance and real partnership has increased dramatically. I enjoyed the discussions during the webinars, and the assignments,

while a researcher participant noted that

Interacting with other course participants was a great benefit of the course. I found it really interesting hearing from other patient partners and learning from everyone in the small groups and in the larger groups as well.

Engagement in online discussions was high throughout all 4 cohorts, with each participant responding at least once to the discussion posts. Participants also shared suggestions for improving the course. Feedback included that it was hard to connect with some small group members; some groups wanted additional opportunities to engage in POR practice.

#### Common barriers and facilitators to POR

Participants highlighted a variety of barriers and facilitators to engaging in POR. Patient barriers were grouped into 6 themes including lack of accessibility/location barriers, lack of opportunities to engage in POR, complex terms/technical language used in POR, lack of time to participate in research, lack of knowledge, skills, and self-efficacy, and lack of compensation. The most common barriers were lack of time (n = 13), lack of knowledge, skills, and self-efficacy (n = 10), and lack of opportunities to engage in research (n = 7). The most common patient identified facilitators were POR training opportunities (n = 13) and opportunities to engage with researchers conducting POR (n = 7). Other facilitators for patients included being compensated for participation, feeling valued by the research team, and having a high level of interest in the research being conducted.

Researcher barriers were grouped into 5 themes: lack of knowledge and self-efficacy to conduct POR; lack of funding/resources; challenges recruiting patient partners; lack of organizational support/buy-in; and lack of time. The most common barriers were lack of time (n = 11), lack of organizational support and buy-in from leadership (n = 8), and lack of funding and resources (n = 7). Researchers identified POR training and mentorship (n = 13) and having organizational support and buy-in from leadership (n = 7) as the most important facilitators for conducting POR. Other facilitators included understanding the importance of POR as well as funding opportunities and mentorship in POR.

## Discussion

The PiR course had a positive impact on both patient and researcher knowledge and self-efficacy. Notably, there were no observed differences in primary outcome scores between patients and researcher participant groups. We posit that the observed increase in knowledge and self-efficacy was due to the process of course co-creation and delivery. In keeping with theories of critical pedagogy, social and transformative learning, our course design and delivery methods leveraged best practices to create and model an inclusive learning climate, which is conducive to meaningful learning [12]. For instance, educational literature suggests that social interactions within a supportive learning context facilitate increased motivation and self-efficacy, offer important opportunities to co-produce knowledge and invite new ways of learning [13–15]. Furthermore, critical pedagogy and transformative learning theories reinforce the importance of designing educational interventions that use real-world experiences, rely on problem-posing education in which learners reflect on different processes rather than being presented with ready-made answers (i.e., banking approach), and encourage problem-based learning [11, 13]. We used these methods when designing and delivering PiR in order to facilitate the co-production of knowledge in a power-sharing dynamic and encouraged learners to critically engage with the content rather than being passive recipients of it [15].

We interpreted the increase in patient knowledge from post-course to 6-months post course to be related to this group’s already high-level of motivation to learn about POR. These participants were already motivated to learn about and engage in POR prior to their participation in the course; therefore, we posit that they continued to be motivated to increase their POR knowledge, and did so, even after the course.

There were no significant improvements in intention to engage in POR. The lack of increase in intentions to engage in POR may be attributed to the already high baseline scores, which remained high immediately after and 6-months post course. Further, there were no significant increases in patients’ use of POR, which suggests that patients did not find more research opportunities after participating in the course. However, researchers did report increases in use of POR. A plausible explanation for why researchers reported an increase in their use of POR while patients in the course did not is that researchers may have increased their use of POR methods in the planning for and designing of studies but did not necessarily engage participants from the course in these efforts. Although researchers highlighted some persistent barriers to implementing POR (i.e., recruiting patients), the data suggest that overall, they felt better equipped to execute and engage in POR projects following the course. The lack of increase in use of POR among patients may also be attributed to the lack of available networks and opportunities to connect researchers and patient partners, which was highlighted by both patients and researchers as a significant barrier to POR. In our study, patients were typically unaware of relevant research organizations or upcoming POR opportunities. The lack of accessible networking opportunities among researchers and patients highlights a barrier to successful POR at a system-level and points to an opportunity to create an easily accessible POR networking platform. This kind of networking infrastructure would facilitate patient notification of available research opportunities and support researchers to connect with a network of patients interested in partnering on research.

Participants in both groups perceived the course to be of high implementation quality and particularly enjoyed participating in the interactive webinars, facilitated discussion boards, and small group PBL assignments.

We posit that the success of our course could be attributed to the co-design and co-delivery of all course components. We anticipate that our working group, composed of patients, researchers and an education specialist allowed individuals to share their unique expertise and led to a course format that was acceptable to both patient and research participants. Finally, our approach aimed to foster social connection between presenters and participants, which may have led to the supportive and collegial environment described by participants.

There are a number of POR studies that highlight the need for high quality, relevant training in POR [30]. When this course was designed and delivered, there were few POR training opportunities available to both patients and researchers. Furthermore, existing POR training opportunities are often not accessible (e.g., available online) or have minimal real-time engagement and opportunities for applied learning and networking. For example, the PACER program [31] is an intensive, applied training for patients to learn how to conduct research; however, given that it is a 2-year program that requires some in-person components, it is not accessible to patients who are not able to commit to that extended period of time. Further, this program does not bring patients and researchers together to collaboratively learn how to do POR. Another example is the SPOR Masterclass [32], which brings together patients, researchers, practitioners, and policy-makers to learn about health research enterprise broadly. PiR differs from this course in that it exclusively targets patients and researchers and aims to nurture their collaborative research capacities. Our study provides a model of a successful training initiative that used principles of co-creation and adult learning theories to equip patients with an understanding of the complexities of the research process while also providing researchers with practical guidance on how to meaningfully engage patient partners in POR [33–35]. The appetite for such training was clear as our team only had to do one recruitment ‘push’ to recruit over 150 participants to fill 4 cohorts. Of note, participants did not have to pay to participate in the course (thereby eliminating a barrier to participation), which likely further facilitated engagement and accessibility. Despite the continued interest in the PiR course, we were unable to sustain its delivery once funding for this project ended; however, some of the patients have partnered on developing and delivering other courses including rapid reviews during the COVID-19 pandemic [36].

Our findings suggest that individual-level training is not sufficient to support widespread implementation of POR in health research. Rather, system-level approaches to develop patient and researcher networks for collaborative research are needed to sustain effective and equitable POR initiatives. Investments in such platforms and resources to facilitate the conduct and participation in POR are warranted. For example, in the United Kingdom, the National Institute for Health Research funds regional partnerships called Applied Research Collaborations, with the purpose of responding to the research priorities of local populations, services and systems [37]. Similarly, in response to the need for system-level POR capacity development, the Canadian Institutes of Health Research have recently established the SPOR National Training Entity to support approaches for systematic training and collaboration in POR [38].

Finally, we recommend that additional research on how to optimize POR training and patient engagement in research be conducted. Specifically, research on how to engage priority populations or populations who face social and systemic barriers to inclusion in research (e.g., accessibility, language, and economic barriers) is needed. Moreover, frameworks to provide research teams with an understanding of power dynamics, implicit biases, and systems of privilege and tokenism are essential for dismantling inequities in POR initiatives [39].

### Limitations and strengths

This study delivered a co-created PiR course that was evaluated both for implementation quality and impact among participants. However, some limitations to this evaluation should be noted. First, we collected self-reported data, which has inherent biases based on participant perspectives of their level of knowledge and self-efficacy. Second, we did not include a comparison group in the study and thus we are unable to definitively conclude that observed changes in outcomes are a result of the course, rather than external factors. Third, sociodemographic data for course participants was not collected and thus we were unable to determine how representative of the researcher and patient populations the cohorts were as compared to the broader populations. Fourth, our course was delivered online; therefore, participants were those who had access to a computer and internet. Additionally, our course was offered in English and required participants to submit an application in writing. These conditions may have precluded individuals who did not speak English, those with limited written literacy and those without access to the required technology from participating in the course. The exploration of additional course delivery formats to improve equity and reach under-represented populations is needed. Lastly, our survey response rate decreased over time. Almost half of participants did not participate in the evaluation and those who continued to participate in surveys over time may have been more engaged and therefore not representative of the entire cohort.

## Conclusion

Process and outcome evaluations collected over a 9-month period indicated that participation in the PiR course increased participants’ knowledge and self-efficacy in POR. This study helps advance the science on POR training by bringing together education theories and best practices within a POR context. This work enhances our understanding of how to design useful and engaging education opportunities to increase patient and researcher capacity in POR. Additional opportunities to create sustainable POR training initiatives and networks to facilitate patient and researcher interactions are needed.

## Supplementary Information

**Additional file 1**. PiR course overview—Fall 2018

**Additional file 2**. PiR evaluation survey—Patient—Post-course timepoint

**Additional file 3**. PiR Evaluation data sources

**Additional file 4**. Organizational coding framework for open-ended survey data

**Additional file 5**. Figures depicting results of primary outcome evaluation for PiR patient (Fig. S1A) and researcher (Fig. S1B) participants

**Additional file 6**. GRIPP2 reporting checklist short form—evaluation of PiR course

## Data Availability

All data sets and supporting materials for this study are located at the KT program, Li Ka Shing Knowledge Institute, St. Michael’s Hospital-Unity Health Toronto and can be provided upon reasonable request.

## Abbreviations

POR: Patient-oriented research
PiR: Partners in research
KT: Knowledge translation
SPOR: Strategy for patient-oriented research
PBLS: Problem-based learning scenario
TDF: Theoretical domains framework
TDM: Total design method

## References

[1] Canadian Institutes of Health Research (CIHR). “Patient Engagement.” [Online] Date accessed: February 10, 2020. https://cihr-irsc.gc.ca/e/41204.html

[2] Domecq JP, Prutsky G, Elraiyah T, Wang Z, Nabhan M, Shippee N (2014). Patient engagement in research: a systematic review. BMC Health Serv Res.

[3] Woolf SH, Zimmerman E, Haley A, Krist AH (2016). Authentic engagement of patients and communities can transform research, practice, and policy. Health Aff (Millwood).

[4] Andrews L, et al. More than just ticking a box … how patient and public involvement improved the research design and funding application for a project to evaluate a cycling intervention for hip osteoarthritis. *Research Involvement and Engagement,* 2015. 10.1186/s40900-015-0013-8 PMC5611566 29062501

[5] Strategy for Patient-Orient Research: Patient Engagement Framework, 2014. https://cihr-irsc.gc.ca/e/48413.html

[6] Shen S, Doyle-Thomas KAR, Beesley L (2017). How and why should we engage parents as co-researchers in health research? A scoping review of current practices. Health Expect.

[7] Mockford C, Staniszewska S, Griffiths F, Herron-Marx S (2012). The impact of patient and public involvement on UK NHS health care: a systematic review. Int J Qual Health Care.

[8] Nilsen ES, Myrhaug HT, Johansen M, Oliver S, Oxman AD (2006). Methods of consumer involvement in developing healthcare policy and research, clinical practice guidelines and patient information material. Cochrane Database Syst Rev.

[9] Oliver S, Clarke-Jones L, Rees R, Milne R, Buchanan P, Gabbay J, Gyte G, Oakley A, Stein K (2004). Involving consumers in research and development agenda setting for the NHS: developing an evidence-based approach. Health Technol Assess.

[10] Fosnot CT, Perry RS, Fosnot CT (1996). Constructivism: a psychological theory of learning. Constructivism: theory, perspectives, and practice.

[11] Kolb DA (1984). Experiential learning: experience as the source of learning and development.

[12] Taylor EW, Laros A (2014). Researching the practice of fostering transformative learning: lessons learned from the study of andragogy. J Transform Educ.

[13] Forsetlund L, Bjørndal A, Rashidian A, Jamtvedt G, O'Brien MA, Wolf F, Davis D, Odgaard-Jensen J, Oxman AD (2009). Continuing education meetings and workshops: effects on professional practice and health care outcomes. Cochrane Database Syst Rev.

[14] Matthews C (2014). Critical pedagogy in health education. Health Educ J.

[15] Conrad R, Donaldson JA. Engaging the online learner: activities and resources for creative instruction. Updated Edition. Jossey-Bass, An Imprint of Wiley. 2004.

[16] Hyatt SE, Lehman RM, Conceição SCO (2017). Motivating and retaining online students: research-based strategies that work. J Coll Stud Retent Res Theory Pract.

[17] Cook DA, Levinson AJ, Garside S, Dupras DM, Erwin PJ, Montori VM (2008). Internet-based learning in the health professions: a meta-analysis. JAMA.

[18] Nirula L, Lowe m, Maharaj s, McLaney E, Parker K, Leslie K. Teaching Excellence: Competencies Model. Centre for Faculty Development: University of Toronto, 2015

[19] Garrison DR, Anderson T, Archer W (2000). Critical inquiry in a text-based environment: computer conferencing in higher education. Internet Higher Educ.

[20] Organization for Economic Co-operation and Development (OECD). Class size and student teacher ratio. Accessed 2016. https://gpseducation.oecd.org/revieweducationpolicies/#!node=41720&filter=all

[21] Kirkpatrick DL, Kirkpatrick JD (2006). Evaluating training programs: the four levels.

[22] Patient Advisors Network: https://www.patientadvisors.ca

[23] Michie S, van Stralen MM, West R (2011). The behaviour change wheel: a new method for characterising and designing behaviour change interventions. Implement Sci.

[24] Cane J, O'Connor D, Michie S (2012). Validation of the theoretical domains framework for use in behaviour change and implementation research. Implement Sci..

[25] Légaré F, Borduas F, Freitas A (2014). Development of a simple 12-item theory-based instrument to assess the impact of continuing professional development on clinical behavioral intentions. PLoS ONE.

[26] Durlak JA, DuPre EP (2008). Implementation matters: a review of research on the influence of implementation on program outcomes and the factors affecting implementation. Am J Community Psychol.

[27] Qualtrics. Copyright © 2020 Qualtrics. Provo, Utah, USA. 2005. https://qualtrics.com

[28] Dillman DA (1978). Mail and telephone surveys: the total design method.

[29] StataCorp. Stata Statistical Software: Release 16. College Station, TX: StataCorp LLC. 2019.

[30] Bell T, Vat LE, McGavin C, Keller M, Getchell L, Rychtera A, Fernandez N (2019). Co-building a patient-oriented research curriculum in Canada. Res Involv Engagem..

[31] Patient and Community Engagement Research (PaCER). https://pacerinnovates.ca/.

[32] Ontario SPOR Support Unit Masterclass: The use and conduct of patient-oriented research. https://ossu.ca/resources/master-class/.

[33] Pecka SL, Kotcherlakota S, Berger AM (2014). Community of inquiry model: advancing distance learning in nurse anesthesia education. AANA J.

[34] Richardson C, Akhtar I, Smith C, Edmondson A, Morris A, Hargreaves J, Rhodes C, Taylor J (2019). Effective involvement: a report on the evaluation of a research awareness training package for public involvement in health research. Res Involv Engagem.

[35] Haesebaert J, Samson I, Lee-Gosselin H, Guay-Bélanger S, Proteau JF, Drouin G, Guimont C, Vigneault L, Poirier A, Sanon PN, Roch G, Poitras MÈ, LeBlanc A, Légaré F (2018). How to engage patients in research and quality improvement in community-based primary care settings: protocol for a participatory action research pilot study. Res Involv Engagem.

[36] COVID-19 Evidence Synthesis. The SPOR evidence alliance website. Accessed July 13, 2021. https://sporevidencealliance.ca/key-activities/covid-19-evidence-synthesis/.

[37] Collaborating in applied health research. National Institute for Health Research website. Accessed July 13, 2021. https://www.nihr.ac.uk/explore-nihr/support/collaborating-in-applied-health-research.htm.

[38] SPOR Capacity Development Initiative. Canadian Institutes of Health Research website. Updated June 1, 2021. Accessed July 13, 2021. https://cihr-irsc.gc.ca/e/51465.html

[39] Etherington N, Rodrigues IB, Giangregorio L (2020). Applying an intersectionality lens to the theoretical domains framework: a tool for thinking about how intersecting social identities and structures of power influence behaviour. BMC Med Res Methodol.

