# Supplementary material for: Evaluation of the partners in research course: a patient and researcher co-created course to build capacity in patient-oriented research
Source: Res Involv Engagem. 2021 Oct 30;7:76. doi: 10.1186/s40900-021-00316-8 (PMC8556807; doi:10.1186/s40900-021-00316-8)
Supplement: Supplementary file 1 — Additional file 1. PiR course overview—Fall 2018 [file 40900_2021_316_MOESM1_ESM.pdf]

Additional File 1: Partners in Research Course Overview – Fall 2018

Date	Webinar	Topic	Learning objectives	Assignment due date
Monday, Oct. 15, 2018<br>9:30am-11:30am (ET)	Webinar #1	What is POR and why is it important?	1. Define Patient Oriented Research (POR)<br>2. Understand the rationale for POR and how it can improve health systems and practices<br>3. Describe the roles of patients and researchers in POR<br>4. Define Patient Reported Outcomes (PRO)	Assignment #0: Who is in your group? (Due October 12)<br><br>Assignment #1: What is POR? (Due October 26)
Monday Oct. 29, 2018<br>9:30am-11:30am (ET)	Webinar #2 (Patient webinar)	How can you engage in POR?	1. Explain the goal of the Canadian health system<br>2. Describe local health networks and how they function<br>3. Discuss important aspects of health research<br>4. Describe various patient engagement activities throughout the research process<br>5. Identify barriers and facilitators to engaging in POR	Assignment #2: What are barriers and facilitators to POR?<br>(Due November 9)
Tuesday Oct. 30, 2018<br>9:30am-11:30am (ET)	Webinar #2 (Researcher webinar)	How can you engage in POR?	1. Define patient engagement in research<br>2. Discuss ethical considerations related to engaging patients as partners in health research<br>3. Identify barriers and facilitators to engaging in POR	
Monday, Nov. 12, 2018<br>9:30am-11:30am (ET)	Webinar #3	How can patients and researchers be collaborative partners in POR?	1. Describe how to effectively collaborate on health research<br>2. Identify respectful and inclusive communication techniques	Assignment #3: What barrier will you research? (Due November 23)
Monday, Nov. 26, 2018<br>9:30am-11:30am (ET)	Webinar #4	What have we learned so far and what will we do next?	1. Describe the roles of patients and researchers in POR<br>2. Review effective collaboration and communication techniques<br>3. Review barriers and facilitators to practicing POR<br>4. Identify the benefits of POR	N/A
