# Supplementary material for: Evaluation of the partners in research course: a patient and researcher co-created course to build capacity in patient-oriented research
Source: Res Involv Engagem. 2021 Oct 30;7:76. doi: 10.1186/s40900-021-00316-8 (PMC8556807; doi:10.1186/s40900-021-00316-8)
Supplement: Supplementary file 2 — Additional file 2. PiR evaluation survey—Patient—Post-course timepoint [file 40900_2021_316_MOESM2_ESM.pdf]

Additional File 2: PiR Evaluation Survey - Patient - Post-course timepoint

Thank you for agreeing to participate in the Partners in Research (PiR) course evaluation!

As a reminder, we are asking for your feedback to:

1. Evaluate course outcomes, i.e., whether the PiR course changes:
   - your knowledge of patient-oriented research
   - confidence in your abilities to practice patient-oriented research (self-efficacy)
   - your intention towards engaging in patient-oriented research
   - your use of patient-oriented research
2. Evaluate the process of the course:
   - Whether the PiR course was delivered with quality
   - Whether you were satisfied by the PiR course offerings
   - To gain general feedback on strengths of the course and areas the course could be improvement

The survey should take no more than 40 minutes to complete.

To start the survey, please enter your participant ID and click on the "next" button, below.

Please enter your participant I.D. in the box below.

____

<u>Section 1: Knowledge and Confidence</u>

In this section, you will be presented with a series of questions and statements related to your knowledge and confidence on topics that will be taught in the PiR course. We understand that participants of this course will have various levels of knowledge with respect to patient-oriented research. These questions and answers will simply be used to help us gauge changes in your knowledge and confidence upon completing the course.

Part 1A) Patient-oriented research concepts

Please rate your level of agreement with each statement on a scale from **strongly disagree** to **strongly agree**.

	I currently have a high level of **knowledge** on this	I currently have a high level of **confidence** in my ability to explain/do this

1. The spectrum of patient engagement	▼ Strongly disagree (1) ... Strongly agree (7)	▼ Strongly disagree (1) ... Strongly agree (7)
2. Why it is important for patients to be involved in research as a partner	▼ Strongly disagree (1) ... Strongly agree (7)	▼ Strongly disagree (1) ... Strongly agree (7)
3. How patient-oriented research can improve health systems and practices	▼ Strongly disagree (1) ... Strongly agree (7)	▼ Strongly disagree (1) ... Strongly agree (7)
4. The role of patients in patient-oriented research	▼ Strongly disagree (1) ... Strongly agree (7)	▼ Strongly disagree (1) ... Strongly agree (7)
5. The role of researchers in patient-oriented research	▼ Strongly disagree (1) ... Strongly agree (7)	▼ Strongly disagree (1) ... Strongly agree (7)

Part 1B) How to engage in patient-oriented research

Please rate your level of agreement with each statement on a scale from **strongly disagree** to **strongly agree**.

	I currently have a high level of **knowledge** on this	I currently have a high level of **confidence** in my ability to explain/do this
1. The stages/life cycle of research	▼ Strongly disagree (1) ... Strongly agree (7)	▼ Strongly disagree (1) ... Strongly agree (7)
2. Who typically conducts research	▼ Strongly disagree (1) ... Strongly agree (7)	▼ Strongly disagree (1) ... Strongly agree (7)
3. How research is funded in the Ontario health system	▼ Strongly disagree (1) ... Strongly agree (7)	▼ Strongly disagree (1) ... Strongly agree (7)
4. Some of the research methods used	▼ Strongly disagree (1) ... Strongly agree (7)	▼ Strongly disagree (1) ... Strongly agree (7)
5. Meaningful vs. tokenistic engagement	▼ Strongly disagree (1) ... Strongly agree (7)	▼ Strongly disagree (1) ... Strongly agree (7)

Part 1C) Effective collaboration and communication techniques

Please rate your level of agreement with each statement on a scale from **strongly disagree** to **strongly agree**.

	I currently have a high level of **knowledge** on this	I currently have a high level of **confidence** in my ability to explain/do this

1. The various collaborators and teams that are involved in patient-oriented research	▼ Strongly disagree (1) ... Strongly agree (7)	▼ Strongly disagree (1) ... Strongly agree (7)
2. How to create roles that allow patients and researchers to work together in the research process	▼ Strongly disagree (1) ... Strongly agree (7)	▼ Strongly disagree (1) ... Strongly agree (7)
3. How to work together to make a decision	▼ Strongly disagree (1) ... Strongly agree (7)	▼ Strongly disagree (1) ... Strongly agree (7)
4. Ways to share my perspectives and experiences	▼ Strongly disagree (1) ... Strongly agree (7)	▼ Strongly disagree (1) ... Strongly agree (7)
5. How to use a common language that everyone can understand	▼ Strongly disagree (1) ... Strongly agree (7)	▼ Strongly disagree (1) ... Strongly agree (7)
6. The importance of active listening	▼ Strongly disagree (1) ... Strongly agree (7)	▼ Strongly disagree (1) ... Strongly agree (7)
7. The experiences that others have had when partnering with researchers on research projects	▼ Strongly disagree (1) ... Strongly agree (7)	▼ Strongly disagree (1) ... Strongly agree (7)

Has your **knowledge** related to patient-oriented research changed since the start of the course? Please include any examples/reasons for this change that you can think of.

Has your **confidence** related to patient-oriented research changed since the start of the course? Please include any examples/reasons for this change that you can think of.

Section 2: Intentions to engage in POR

In this section, you are presented with a series of statements on your **intention** to engage in patient-oriented research.

Intentions
The following questions are related to your <u>intention</u> towards engaging patient-oriented research. Please rate your level of agreement with each statement on a scale from **strongly disagree** to **strongly agree**.

	Strongly disagree (1)	Strongly agree (7)
1. Engaging in patient-oriented research is beneficial	○	○
2. Engaging in patient-oriented research is good	○	○
3. Engaging in patient-oriented research is helpful	○	○
4. People who are important to me think that I should engage in patient-oriented research	○	○
5. I am confident that I could engage in patient-oriented research	○	○
6. For me, conducting patient-oriented research would be easy	○	○
7. I expect to engage in patient-oriented research	○	○
8. I intend to engage in patient-oriented research	○	○

9. Have your **intentions** related to patient-oriented research changed since the start of the course? Please include any examples/reasons for this change that you can think of.

<u>Section 3: Use of POR</u>
In this section, you are presented with a series of statements related to your current **engagement in POR**.

Your current behaviours
Please rate your level of agreement with each statement on a scale from **strongly disagree** to **strongly agree**.

	Strongly disagree (1)	Strongly agree (7)
1. I engage/have engaged with researchers in patient-oriented research.	○	○
2. I take/have taken part in a meaningful conversation about patient-oriented research, including developing a research project.	○	○
3. I engage/have engaged in patient-oriented research.	○	○

4. How have you engaged in meaningful patient-oriented research with research partners? Please describe in a few sentences or list a few examples.

5. What are your **barriers** to engaging in patient-oriented research?

6. What are your **facilitators** to engaging in patient-oriented research?

<u>Section 4: Course Feedback</u>
In this section, you are presented with a series of statements on your satisfaction related to aspects of the PiR course.

4A) Course format

Please rate your level of agreement with each statement on a scale from strongly disagree to strongly agree.

	Strongly disagree (15)	Strongly agree (21)
1. Overall, I enjoyed learning through the online webinars	○	○
2. Overall, I thought the online webinars were effective in delivering educational sessions	○	○
3. I liked interacting with people on the online learning platform	○	○
4. I liked interacting with people in group assignments	○	○
5. I liked the frequency of the webinars	○	○
6. Overall, the resources on the online learning platform were easy to access	○	○

4B) Course materials

Please rate your level of agreement with each statement on a scale from strongly disagree to strongly agree.

	Strongly disagree (15)	Strongly agree (21)
1. The resources provided were helpful in my learning	○	○
2. The group discussions were helpful in my learning	○	○
3. The online webinar activities (e.g., polling activities) were relevant to the overall content of the course.	○	○
4. The online webinar activities (e.g., polling activities) were helpful in my learning	○	○
5. The post-webinar assignments (e.g., group assignments) were related to were relevant to the overall content of the course	○	○
6. The post-webinar assignments were helpful in learning more about patient-oriented research	○	○
7. The online quizzes were helpful for my learning	○	○

8. There were multiple resources used in the PiR course, for example the readings, the videos, blogs, etc. Please select which of the following you have used:

- [ ] Readings
- [ ] Videos
- [ ] Blogs
- [ ] Case studies
- [ ] Presentation slides
- [ ] Contacts/organizations
- [ ] PiR activities
- [ ] PiR Discussion board

4C) Quality of delivery

Please rate your level of agreement with each statement on a scale from strongly disagree to strongly agree.

	Strongly disagree (15)	Strongly agree (21)
1. The learning objectives were clearly outlined	○	○
2. The learning objectives were regularly met	○	○
3. The webinars were engaging	○	○
4. The group discussions were engaging	○	○
5. The content was relevant to me	○	○
6. I would recommend this course to other people	○	○
7. The course was delivered with high quality	○	○
8. The presenters were engaging and informative	○	○
9. The course facilitators supported me in my learning	○	○

4D) Participants

Please rate your level of agreement with each statement on a scale from strongly disagree to strongly agree.

	Strongly disagree (15)	Strongly agree (21)
1. The course created a sense of community	○	○
2. I found that I learned from other participants	○	○
3. I created a relationship with other participants	○	○

4E) Satisfaction with course

In this section, you are presented with a series of statements related to the implementation quality of the course as well as your satisfaction with the course components. Rate your level of satisfaction with the following aspects of the course below from extremely dissatisfied to extremely satisfied.

	Extremely dissatisfied (30)	Extremely satisfied (36)
1. Course resources	○	○
2. Presentations	○	○
3. How the content applies/relates to me	○	○
4. Session activities	○	○
5. The format of the webinar sessions	○	○
6. The content of the sessions	○	○
7. Online webinar activities (e.g., polling activities)	○	○
8. Post-webinar assignments	○	○
9. The course overall	○	○

Section 5: General Course Feedback

In this section, you are asked to provide feedback regarding the things you liked about the course as well as things that could be improved upon.

Which aspect of the course had the biggest impact on you, if any?

Please explain what parts of the course you enjoyed:

Please explain what parts of the course could be improved:

Were there any components of the course that didn't work for you?

How has the course compared to other capacity building workshops you have done?

Section 6: Next steps

Thank you for completing the PiR evaluation survey. We truly value the information you have provided. If you have any additional comments to include, please feel free to add them below.
