# Supplementary material for: Evaluation of the partners in research course: a patient and researcher co-created course to build capacity in patient-oriented research
Source: Res Involv Engagem. 2021 Oct 30;7:76. doi: 10.1186/s40900-021-00316-8 (PMC8556807; doi:10.1186/s40900-021-00316-8)
Supplement: Supplementary file 3 — Additional file 3. PiR Evaluation data sources [file 40900_2021_316_MOESM3_ESM.pdf]

Additional File 3: Partners in Research Evaluation Data Sources

<table>
<tr><th>Outcomes</th><th>Indicator</th><th>PiR Indicators</th><th>Data source</th></tr>
<tr><td>Primary:
Impact of course on participant knowledge, self-efficacy, intentions, use of POR</td><td>Self-reported change in:
• Knowledge
• Self-efficacy
• Intentions
• Use of POR</td><td>• Self-identified knowledge of POR concepts
• Self-identified self-efficacy to engage in POR
• Self-identified intentions to engage in POR
• Self-reported engagement in POR</td><td>• Quantitative and qualitative survey data from 3 time-points (baseline, post-course, 6 months post-course)</td></tr>
<tr><td rowspan="3">Secondary outcome:
Quality of implementing the course</td><td>Reach</td><td>• # of participants in each cohort</td><td>• Course administrative documents</td></tr>
<tr><td>Dose:</td><td>• # of webinars delivered
• # of discussion questions posed by coaches</td><td>• Course admin records
• Online discussion boards</td></tr>
<tr><td>Participant responsiveness</td><td>• # of responses on discussion board
• Participant self-reported course satisfaction</td><td>• Online discussion boards
• Quantitative and qualitative survey data from 2nd time-point (post-course survey)</td></tr>
<tr><td>Secondary outcome:
Identify barriers and facilitators to engaging in POR</td><td>Barriers and facilitators to engaging in POR</td><td>• Self-identified barriers and facilitators to engaging in POR</td><td>• Qualitative survey data from 3 time-points (baseline, post-course, 6 months post-course)</td></tr>
</table>

Abbreviations: POR, patient-oriented research
