# Supplementary material for: Evaluation of the partners in research course: a patient and researcher co-created course to build capacity in patient-oriented research
Source: Res Involv Engagem. 2021 Oct 30;7:76. doi: 10.1186/s40900-021-00316-8 (PMC8556807; doi:10.1186/s40900-021-00316-8)
Supplement: Supplementary file 4 — Additional file 4. Organizational coding framework for open-ended survey data [file 40900_2021_316_MOESM4_ESM.pdf]

Additional File 4: Organizational Coding Framework for Open-Ended Survey Data

Outcome	Construct	Time-point	Comments
**Primary**	Knowledge	• Baseline<br>• Post-course<br>• 6 month post-course	This node will capture participant awareness of POR concepts and self-reported changes in knowledge
	Self-efficacy (beliefs about capabilities)	• Baseline<br>• Post-course<br>• 6 month post-course	This node will capture participant self-efficacy in engaging in POR and self-reported changes in self-efficacy
	Intentions	• Baseline<br>• Post-course<br>• 6 month post-course	This node will capture participant intentions to engage in POR and self-reported changes in intentions
	Behaviour	• Baseline<br>• Post-course<br>• 6 month post-course	This node will capture participant behavior to use POR concepts and self-reported changes in behaviour
**Secondary**	Implementation quality	• Post-course	This node will capture participant perceptions on course quality and delivery
	Participant responsiveness	• Post-course	This node will capture participant perceptions on course content, structure, engagement and resources
	Barriers and facilitators to engagement in POR	• Baseline<br>• Post-course<br>• 6 month post-course	This node will capture actual and anticipated participant identified barriers and facilitators to engagement in POR.
