# Supplementary material for: Evaluation of the partners in research course: a patient and researcher co-created course to build capacity in patient-oriented research
Source: Res Involv Engagem. 2021 Oct 30;7:76. doi: 10.1186/s40900-021-00316-8 (PMC8556807; doi:10.1186/s40900-021-00316-8)

Additional File 5: Figures depicting results of primary outcome evaluation for PiR patient (Figure 1A) and researcher (Figure 1B) participants

Figure 1A. Mean score of **PiR patient** course participants primary outcomes (knowledge, self-efficacy, intentions and use of POR) out of a 7-point likert scale (*error bars indicate standard deviation)*

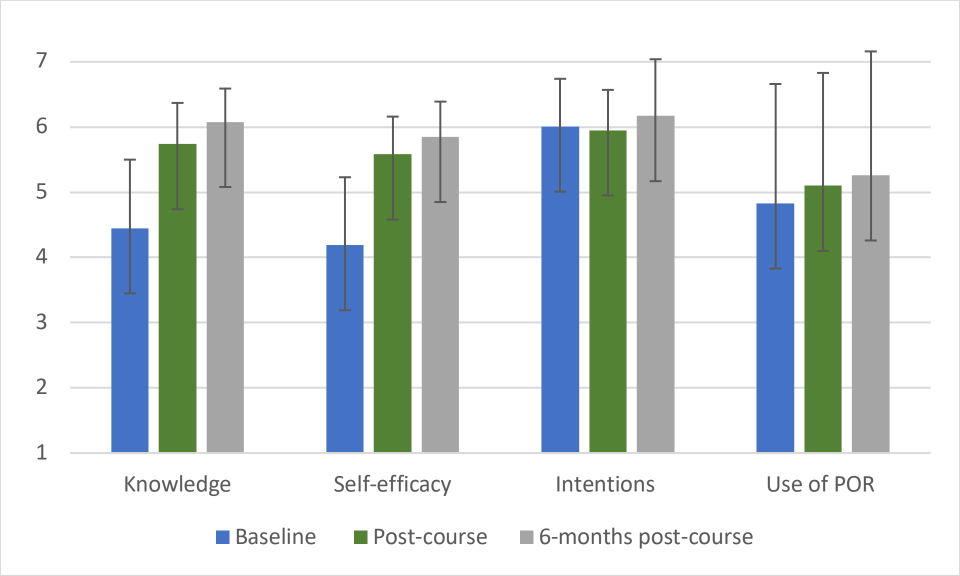

Figure 1B. Mean score of **PiR researcher** course participants primary outcomes (knowledge, self-efficacy, intentions and use of POR) out of a 7-point likert scale (*error bars indicate standard deviation)*

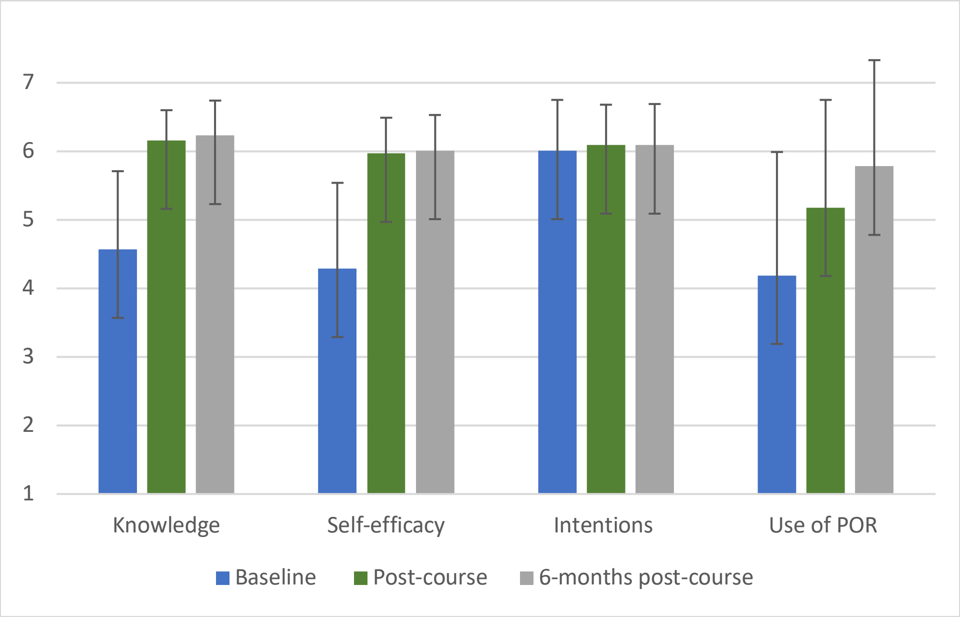

Supplement: Supplementary file 5 — Additional file 5. Figures depicting results of primary outcome evaluation for PiR patient (Fig. S1A) and researcher (Fig. S1B) participants [file 40900_2021_316_MOESM5_ESM.pdf]
