# Supplementary material for: Evaluation of the partners in research course: a patient and researcher co-created course to build capacity in patient-oriented research
Source: Res Involv Engagem. 2021 Oct 30;7:76. doi: 10.1186/s40900-021-00316-8 (PMC8556807; doi:10.1186/s40900-021-00316-8)
Supplement: Supplementary file 6 — Additional file 6. GRIPP2 reporting checklist short form—evaluation of PiR course [file 40900_2021_316_MOESM6_ESM.pdf]

Additional file 6: GRIPP2 reporting checklist short form – Evaluation of PiR Course

Section and topic	Item	Reported on page No
1: Aim	Report the aim of PPI in the study	pp. 5-6
2: Methods	Provide a clear description of the methods used for PPI in the study	pp. 6-7
3: Study results	Outcomes—Report the results of PPI in the study, including both positive and negative outcomes	pp. 12-17
4: Discussion and conclusions	Outcomes—Comment on the extent to which PPI influenced the study overall. Describe positive and negative effects	pp. 18-20
5: Reflections/critical perspective	Comment critically on the study, reflecting on the things that went well and those that did not, so others can learn from this experience	pp. 20-21

Abbreviations: *PPI* patient and public involvement
